# NVP-BKM120 inhibits colon cancer growth via FoxO3a-dependent PUMA induction

**DOI:** 10.18632/oncotarget.20943

**Published:** 2017-09-15

**Authors:** Shida Yang, Xin Li, Wenchang Guan, Mingqin Qian, Zhicheng Yao, Xiaoxue Yin, Hongmei Zhao

**Affiliations:** ^1^ Department of Laboratory Medicine, The People’s Hospital of Liaoning Province, Shenyang, China; ^2^ Department of Anesthesia, The People’s Hospital of Liaoning Province, Shenyang, China; ^3^ Department of Gynaecology and Obstetrics, The People’s Hospital of Liaoning Province, Shenyang, China; ^4^ Department of Ultrasound Diagnosis, The People’s Hospital of Liaoning Province, Shenyang, China; ^5^ Department of Neurology, The People’s Hospital of Liaoning Province, Shenyang, China

**Keywords:** NVP-BKM120, PUMA, apoptosis, FoxO3a, colon cancer

## Abstract

NVP-BKM120, a potent and highly selective PI3K inhibitor, is currently being investigated in phase I/II clinical trials. The mechanisms of action of NVP-BKM120 in colon cancer cells are unclear. In the present study, we investigated how NVP-BKM120 suppresses colon cancer cells growth and potentiates effects of other chemotherapeutic drugs. We found that NVP-BKM120 treatment enhance PUMA induction irrespective of p53 status through the FoxO3a pathway following AKT inhibition. Furthermore, PUMA is required for NVP-BKM120-induced apoptosis in colon cancer cells. In addition, NVP-BKM120 also synergized with 5-Fluorouracil or regorafenib to induce marked apoptosis via PUMA induction. Deficiency of PUMA suppressed apoptosis and antitumor effect of NVP-BKM120 in xenograft model. These results demonstrate a key role of PUMA in mediating the anticancer effects of NVP-BKM120 and suggest that PUMA could be used as an indicator of NVP-BKM120 sensitivity, and also have important implications for it clinical applications.

## INTRODUCTION

Phosphatidylinositol-4,5-bisphosphate 3-kinases (PI3Ks) are belong to a family of enzymes, which are response to growth, cell proliferation, apoptosis, angiogenesis, DNA repair, motility, differentiation and survival [1, 2]. In cell signaling pathway, PI3Ks function as intermediate signaling molecules including PI3K/AKT/mTOR signaling pathway [1, 3, 4]. NVP-BKM120 (Buparlisib) is a small molecule orally-available compound, which has potent pan-class I PI3K inhibitory capability against the p110α/β/δ/γ catalytic subunit isoforms [5, 6]. Previously studies demonstrated that NVP-BKM120 exerts anti-proliferative and cytotoxic effects on solid tumor and hematological malignancies via selective AKT inhibition [7]. Several recent reports also demonstrated that NVP-BKM120 combine with other signaling pathways inhibitors promote its antitumor effects in mouse models [8-10].

P53 upregulated modulator of apoptosis (PUMA, also known as BBC3) belongs to proapototic protein, member of BH3-only Bcl-2 protein family. In cancer cells, PUMA play a key role in apoptosis regulation [11, 12]. PUMA can be induced by p53 -dependent and -independent manner. In p53-dependent transcription manner, DNA damage agents, such as common chemotherapeutic drugs, γ-irradiation, activate p53 and initiate apoptosis through PUMA induction [13]. Moreover, PUMA induction via p53-independent manner is modulated by the transcription factor such as p73, FoxO3a, E2F1, STAT1, or NF-κB [14, 15]. PUMA induction potently promotes apoptosis in cancer cells by binding to antiapoptotic Bcl-2 family members (Bcl-2 and Bcl-XL), which activates the proapoptotic Bcl-2 family members (Bax and Bak), resulting in dysfunction of mitochondrial leading to caspase cascade activity [16].

In this study, our results indicate that NVP-BKM120-induced PUMA induction through the AKT/FoxO3a pathway, and PUMA plays a key role in therapeutic response to NVP-BKM120 in CRC. Our results suggest that PUMA induction is indicative of the therapeutic efficacy of NVP-BKM120.

## RESULTS

### NVP-BKM120 induces apoptosis in CRC

**Figure 1:**
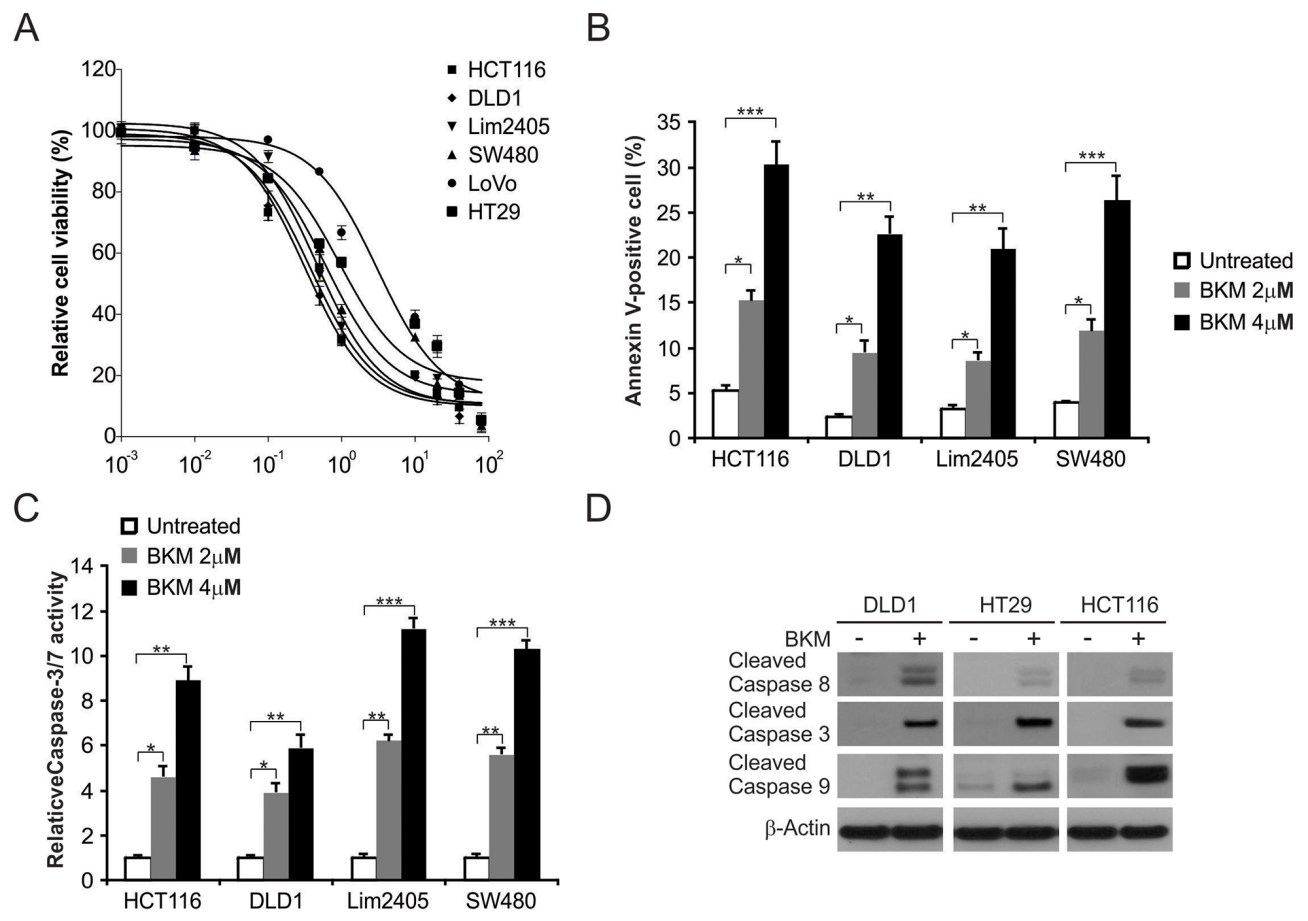
NVP-BKM120 induces apoptosis in CRC **(A)** The indicated cell lines were treated with increasing dose of NVP-BKM120 for 72 hours. Cell proliferation was determined by MTS assay. **(B)** The indicated cell lines were treated with increasing NVP-BKM120 for 24 hours at indicated concentrations. Apoptosis was analyzed by Annexin V/PI staining followed by flow cytometry. **(C)** The indicated cell lines were treated with NVP-BKM120 for 24 hours at indicated concentrations. Caspase 3/7 activity was determined by fluorogenic analysis. **(D)** The indicated cell lines were treated with 4μmol/LNVP-BKM120 for 24 hours. Cleaved caspase 3, 8 and 9 were analyzed by Western blotting. Results in (A), (B) and (C) were expressed as means ± SD of 3 independent experiments. ***, *P*<0.001;**, *P*<0.01; *, *P*<0.05.

To investigate the effective of NVP-BKM120 on CRC, six colon cancer cells were treated with different doses of NVP-BKM120 for 72 hours. MTS analysis was performed to quantify cell proliferation. Indeed, treatment of these cells with NVP-BKM120 led to decreased cell growth (Figure 1A). Apoptosis in HCT116, DLD1, Lim2405 and SW480 cells treated with NVP-BKM120 was analyzed by flow cytometry. As shown in Figure 1B, NVP-BKM120 increased does-dependent Annexin V-positive cells in these cell lines. Furthermore, it has been shown that NVP-BKM120 also induces caspases 3/7 activation in these cell lines (Figure 1C). In addition, HCT116, DLD1 and HT29 were treated with NVP-BKM120 for 24 hours, and then caspase activation was analyzed by Western blotting. As shown in Figure 1D, NVP-BKM120 induced caspase 3, 8 and 9 activation in these cells. These results suggest that NVP-BKM120 decreased cell proliferation and contributed to caspase-dependent apoptosis induction in CRC cells.

### NVP-BKM120 induces p53-independent PUMA upregulation in CRC

**Figure 2:**
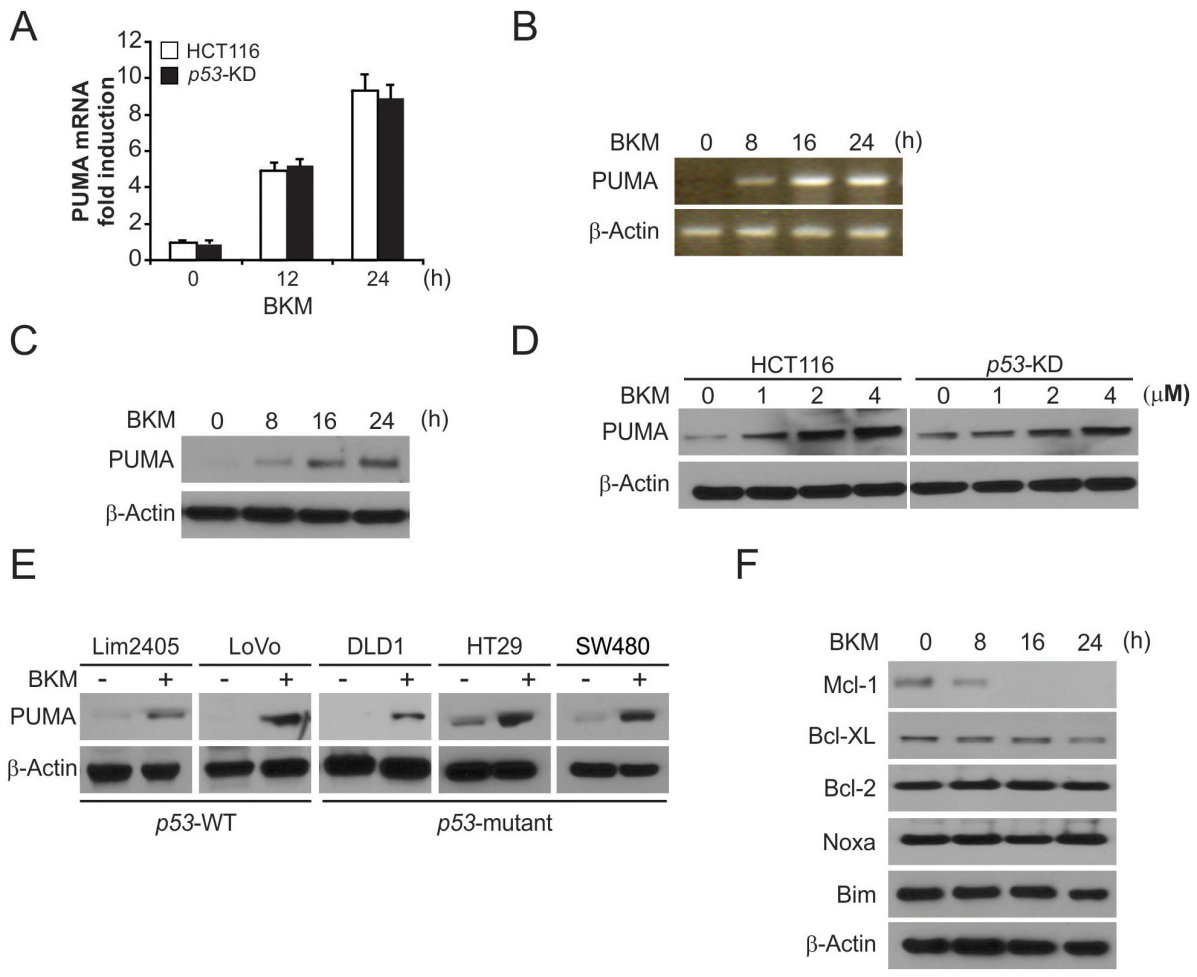
NVP-BKM120 induces p53-independent PUMA induction **(A)** Parental and *p53*-KD HCT116 cells were treated with NVP-BKM120 at indicated time point. *PUMA* mRNA induction by NVP-BKM120 was analyzed by real-time reverse transcriptase (RT) PCR, with *β-actin* as a control. **(B)** HCT116 cells were treated with 4 μmol/L NVP-BKM120 at indicated time point. Total RNA was extracted, and *PUMA* mRNA expression was analyzed by semiquantitive reverse transcription PCR (RT-PCR). β-actin was used as a control. **(C)** HCT116 cells were treated with 4 μmol/L NVP-BKM120 at indicated time point. PUMA expression was analyzed by Western blotting. **(D)** Parental and *p53*-KD HCT116 cells were treated with NVP-BKM120 for 24 hours at indicated concentration. PUMA expression was analyzed by Western blotting. **(E)** Indicated colon cancer cell lines with different *p53* status were treated with 4 μmol/L NVP-BKM120 for 24 hours. PUMA expression was analyzed by Western blotting. **(F)** HCT116 cells were treated with 4 μmol/L NVP-BKM120 at indicated time point. Indicated protein expression was analyzed by Western blotting.

We then analyzed the mechanism of NVP-BKM120 induced apoptosis in CRC cells. Treating HCT116 cells with 4 μmol/L NVP-BKM120 significantly increased the protein and mRNA level of PUMA in a time-dependent manner (Figure 2A-2C). NVP-BKM120 also increased PUMA protein and mRNA level in *p53*-Knockdown (*p53*-KD) HCT116 cells [17] (Figure 2A and 2D). Moreover, NVP-BKM120 upregulated PUMA expression in *p53*-WT Lim2405, LoVo CRC cells, as well as *p53*-mutant DLD1, HT29 and SW480 CRC cells (Figure 2E). In addition, as shown in Figure 2F, NVP-BKM120 treatment did not increased Bim and Noxa protein level, but decreased the anti-apoptotic Mcl-1 level. The above results demonstrate that PUMA is selective upregulated by NVP-BKM120 regardless of p53 status and may mediate its antitumor activity.

### PUMA mediates the anticancer effect of NVP-BKM120

**Figure 3:**
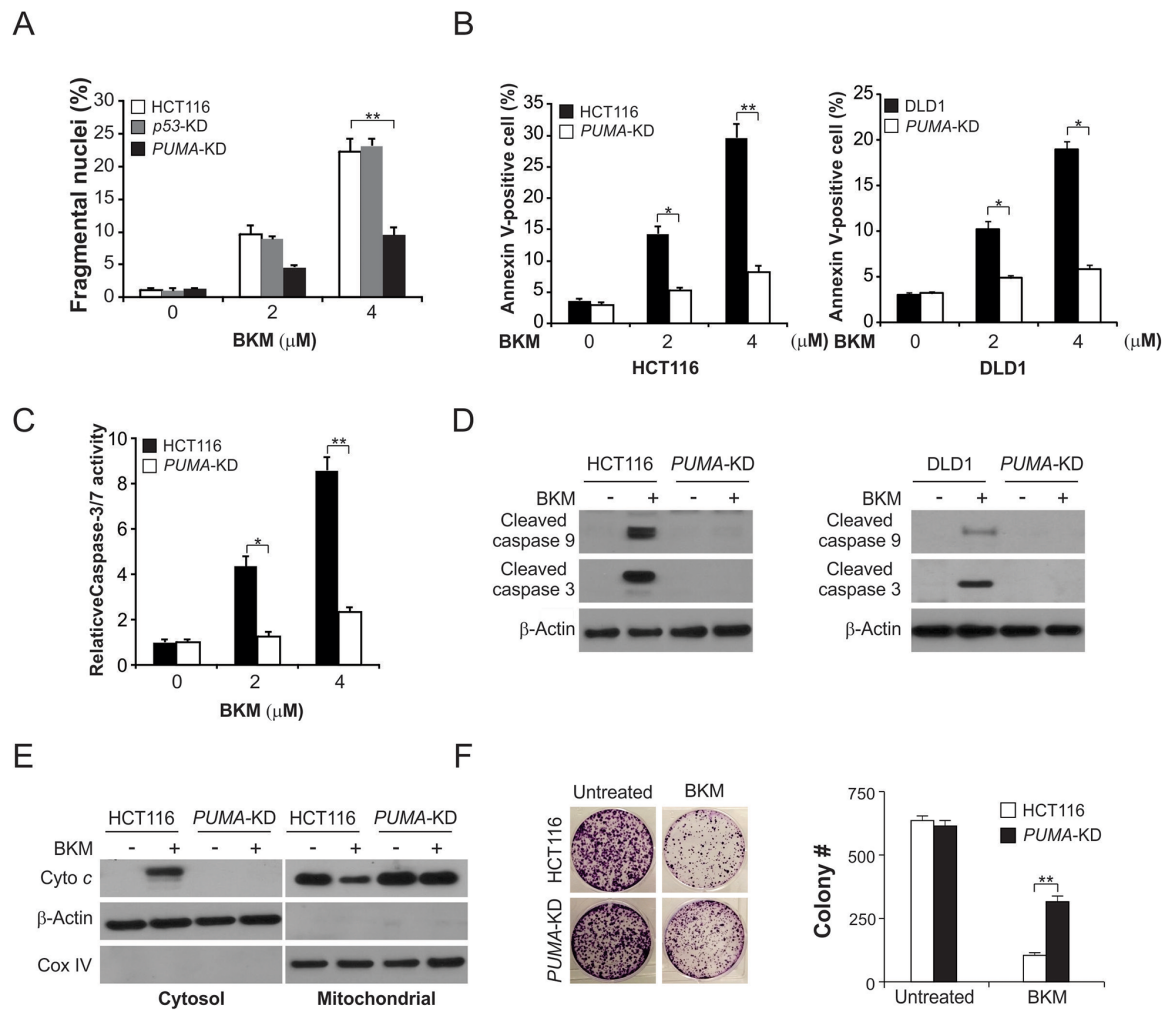
PUMA is required for the apoptotic activity of NVP-BKM120 **(A)** Parental, *p53*-KD and *PUMA*-KD HCT116 cells were treated with NVP-BKM120 at indicated concentration for 24 hours. Apoptosis was analyzed by a nuclear fragmentation assay. **(B)** Parental and *PUMA*-KD HCT116 or DLD1 cells were treated with NVP-BKM120 at indicated concentration for 24 hours. Apoptosis was analyzed by Annexin V/PI staining followed by flow cytometry. **(C)** Parental and *PUMA-*KO *HCT116* cells were treated with NVP-BKM120 for 24 hours. Caspase 3/7 activity was determined by fluorogenic analysis. **(D)** Parental and *PUMA*-KD HCT116 or DLD1 cells were treated with 4 μmol/L NVP-BKM120 for 24 hours. Cleaved caspase 3 and 9 were analyzed by Western blotting. **(E)** The cytoplasm and mitochondria were fractionated from parental and *PUMA-*KO *HCT116* cells treated with 4 μmol/L NVP-BKM120 for 24 hours. The distribution of cytochrome *c* was analyzed by Western blotting. β-Actin and cytochrome oxidase subunit IV (Cox IV) were analyzed as the control for loading and fractionation. **(F)** Parental and *PUMA*-KD HCT116 cells were treated with 4 μmol/L NVP-BKM120 for 24 hours. Colony formation assay was done by seeding an equal number of treated cells in 12-well plates, and then staining attached cells with crystal violet 14 days later. *Left*, representative pictures of colonies; *Right*, quantification of colony numbers. Results in (A), (B), (C) and (F) were expressed as means ± SD of 3 independent experiments.**, *P*<0.01; *, *P*<0.05.

Furthermore, to examine the function of PUMA in NVP-BKM120-induced apoptosis, we generated PUMA stable knockdown (*PUMA*-KD) HCT116 cells [17]. In *PUMA*-KD cells, apoptosis was significantly reduced, which was induced by 2-4 μmol/L NVP-BKM120 (Figure 3A). The reduction of NVP-BKM120-induced apoptosis in *PUMA*-KD HCT116 and DLD1 cells were confirmed by flow cytometry analysis following Annexin V/PI staining (Figure 3B). Consistently, the activation of caspase 3/7, which induced by NVP-BKM120, was reduced in *PUMA*-KD HCT116 cells (Figure 3C). The deficiency of PUMA abrogated NVP-BKM120-induced mitochondrial pathway apoptosis, which was observed in cleaved caspases 3 and 9 in HCT116 and DLD1 cells (Figure 3D) and cytochrome *c* release in HCT116 cells (Figure 3E). Notably, in the long-term colony formation assay, compare with parental cells, *PUMA*-KD cells had improved survival following NVP-BKM120 treatment (Figure 3F). These results indicate that apoptotic response of NVP-BKM120 is PUMA-dependent in CRC.

### FoxO3A is involved in NVP-BKM120 induced PUMA up-regulation

**Figure 4:**
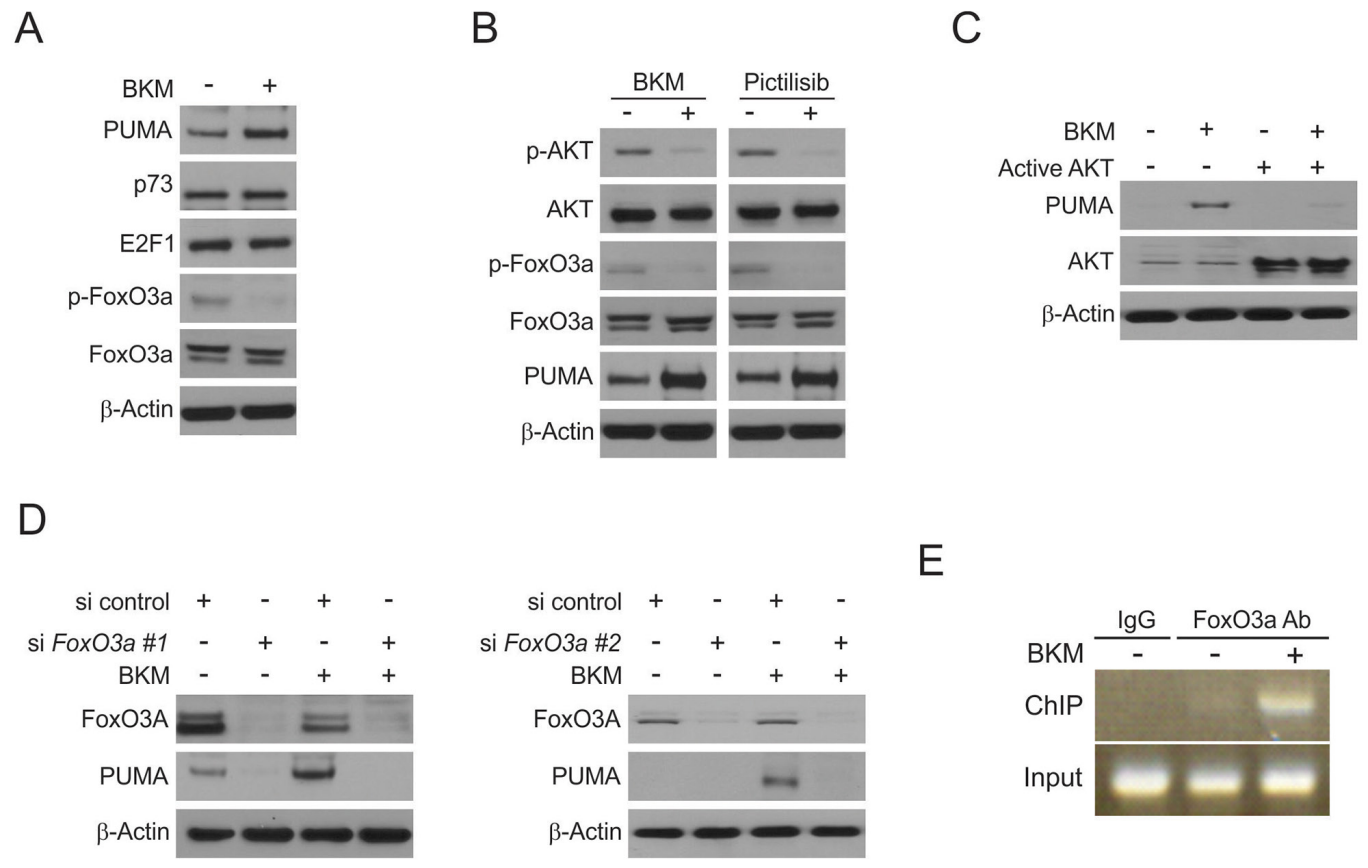
Induction of PUMA by NVP-BKM120 is mediated through the AKT inhibition **(A)** HCT116 cells were treated with 4μmol/L NVP-BKM120 for 24 hours. PUMA, p73, E2F1, p-FoxO3a and FoxO3a expression was analyzed by Western blotting. **(B)** HCT116 cells were treated with 4μmol/L NVP-BKM120 or 4μmol/L Pictilisib for 24 hours. Indicated protein levels were analyzed by Western blotting. **(C)** HCT116 cells were transfected with active AKT plasmid for 6 hours, and then treated with 4μmol/L NVP-BKM120 for 24 hours. PUMA and AKT expression was analyzed by Western blotting. **(D)** HCT116 cells were transfected with either a control scrambled siRNA or *FoxO3a* siRNA for 24 hours, and then treated with 4μmol/L NVP-BKM120 for 24 hours. FoxO3a and PUMA expression was analyzed by Western blotting. siRNA FoxO3a #1 from Santa Cruz Biotechnology and siRNA FoxO3a #2 Thermo Fisher Scientific. **(E)** Chromatin immunoprecipitation (ChIP) was performed using anti-FoxO3a antibody on HCT116 cells following NVP-BKM120 treatment for 12 hours. ChIP with the control IgG was used as a control. PCR was carried out using primers surrounding the FoxO3a binding sites in the *PUMA* promoter.

To further explore the mechanism of PUMA induction following NVP-BKM120 treatment in CRC cells, several transcription factors that can induce PUMA expression in *p53*-KD cells were examined. We exclude p73 and E2F1 due to lack of induction (Figure 4A). Interestingly, NVP-BKM120 treatment markedly dephosphorylation AKT at S473, also reduced FoxO3a phosphorylation, which is known to prevent its nuclear translocation and ensuing transactivation (Figure 4A and 4B). PUMA induction was suppressed by exogenous expression of active AKT following NVP-BKM120 treatment (Figure 4C). Furthermore, NVP-BKM120-induced PUMA upregulation was abrogated by FoxO3a knockdown in HCT116 cells (Figure 4D). Moreover, treating cells with pictilisib, which is another AKT inhibitor, was sufficient to induce the up-regulation of PUMA following AKT and FoxO3a dephosphorylation (Figure 4B). To further investigate if FoxO3a can directly binding to *PUMA* promoter, chromatin immunoprecipitation (ChIP) was performed. FoxO3a was found to be recruited to PUMA promoter following NVP-BKM120 treatment (Figure 4E). Together, these data suggest that in response to NVP-BKM120 treatment, FoxO3a directly binds to *PUMA* promoter in order to drive it transcriptional activation.

### PUMA mediates the chemo-sensitization effects of NVP-BKM120

**Figure 5:**
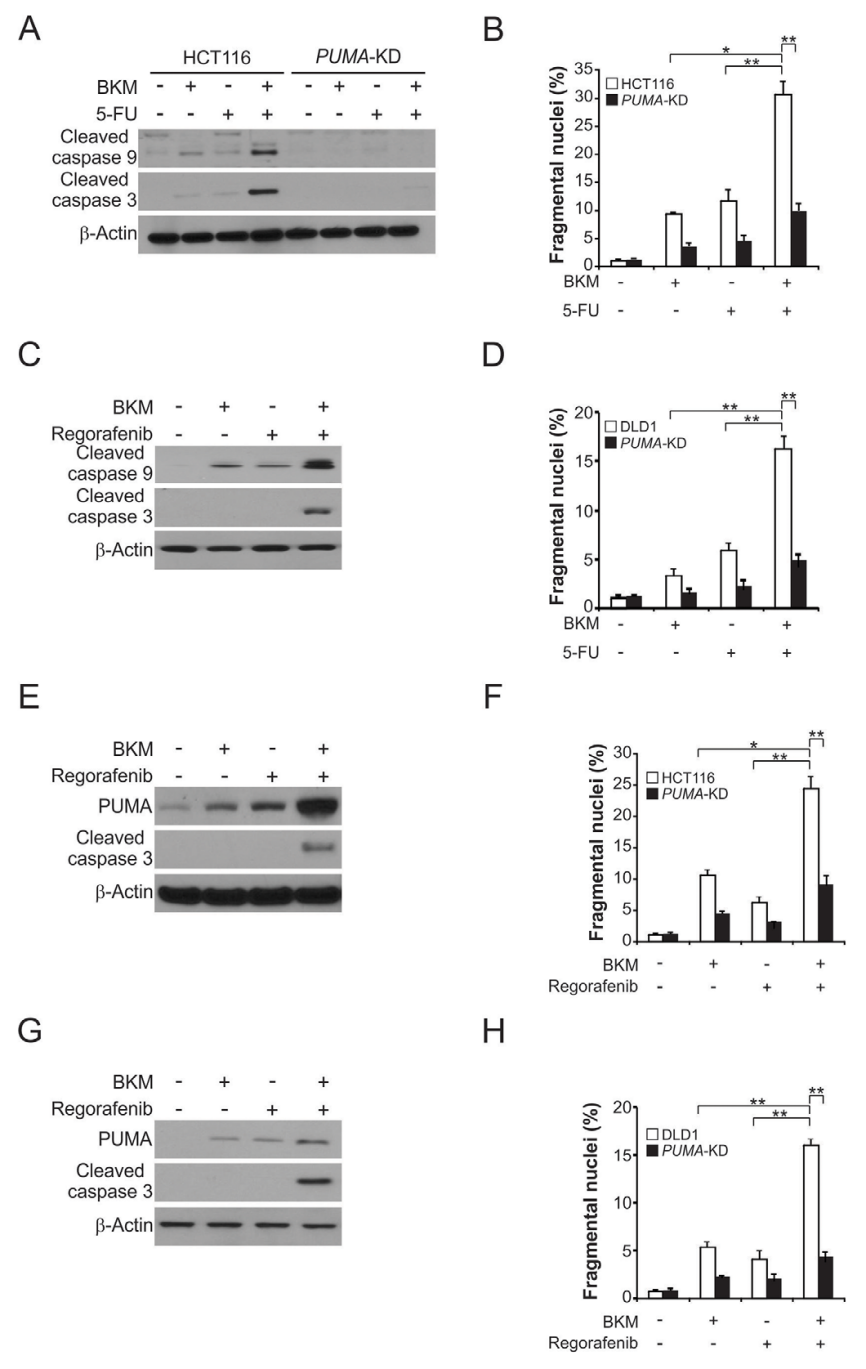
NVP-BKM120 synergizes with 5-FU or regorafenib to induce apoptosis via PUMA in CRC **(A)** Parental and *PUMA*-KD HCT116 cells were treated with 2 μmol/L NVP-BKM120, 20 mg/L 5-fluorouracil (5-FU), or their combination for 24 hours. Cleaved caspase 3 and 9 were analyzed by Western blotting. **(B)** Parental and *PUMA*-KD HCT116 cells were treated 2 μmol/L NVP-BKM120, 20 mg/L 5-FU, or their combination for 24 hours. Apoptosis was analyzed by a nuclear fragmentation assay. **(C)** Parental and *PUMA*-KD DLD1 cells were treated with 2 μmol/L NVP-BKM120, 20 mg/L 5-FU or their combination for 24 hours. Cleaved caspase 3 and 9 were analyzed by Western blotting. **(D)** Parental and *PUMA*-KD DLD1 cells were treated 2 μmol/L NVP-BKM120, 20 mg/L 5-FU, or their combination for 24 hours. Apoptosis was analyzed by a nuclear fragmentation assay. **(E)** HCT116 cells were treated with 2 μmol/L NVP-BKM120, μmol/L regorafenib, or their combination for 24 hours. PUMA and cleaved caspase 3 were analyzed by Western blotting. **(F)** Parental and *PUMA*-KD HCT116 cells were treated 2 μmol/L NVP-BKM120, 20 μmol/L regorafenib, or their combination for 24 hours. Apoptosis was analyzed by a nuclear fragmentation assay. **(G)** DLD1 cells were treated with 2 μmol/L NVP-BKM120, 20 μmol/L regorafenib, or their combination for 24 hours. PUMA and cleaved caspase 3 were analyzed by Western blotting. **(H)** Parental and *PUMA*-KD DLD1 cells were treated 2 μmol/L NVP-BKM120, 20 μmol/Lregorafenib, or their combination for 24 hours. Apoptosis was analyzed by a nuclear fragmentation assay. Results in (B), (D), (F) and (H) were expressed as means ± SD of 3 independent experiments. **, *P*<0.01; *, *P*<0.05.

NVP-BKM120 has been used in combination with other chemotherapeutic agents [18]. While the mechanism by which the chemo-sensitization effects of NVP-BKM120 is not well understood. From the above results, we reasoned that the chemo-sensitization effects of NVP-BKM120 were mediated by PUMA induction, due to concurrent PUMA induction following NVP-BKM120 and other agent treatment through different pathways. Importantly, we found that higher levels of PUMA was stimulated by NVP-BKM120 combined with 5-FU, compared to single agent alone treatment (Figure 5A). The result is consistent with parallel PUMA induction via *p53*-dependent and -independent mechanism by DNA damage and NVP-BKM120. Following the combination treatment, the apoptosis level and caspase 3 and 9 activation were markedly increased in parental cells, in contrary to *PUMA*-KD HCT116 and DLD1 cells (Figure 5A-5D). Furthermore, when HCT116 or DLD1 cells were treated with NVP-BKM120 and regorafenib, the PUMA-dependent sensitization effect was also detected (Figure 5E and 5G). Accordingly, we found that the combination treatment promote the level of apoptosis as well as caspase 3 activation, but not in *PUMA*-KD cells (Figure 5E-5H). These findings demonstrate that therapeutic efficacy of NVP-BKM120 can be markedly improved by PUMA-dependent chemo-sensitization effects of NVP-BKM120, and manipulating PUMA-mediated apoptosis.

### PUMA contributes to the antitumor activity of NVP-BKM120 in a mouse xenograft model

**Figure 6:**
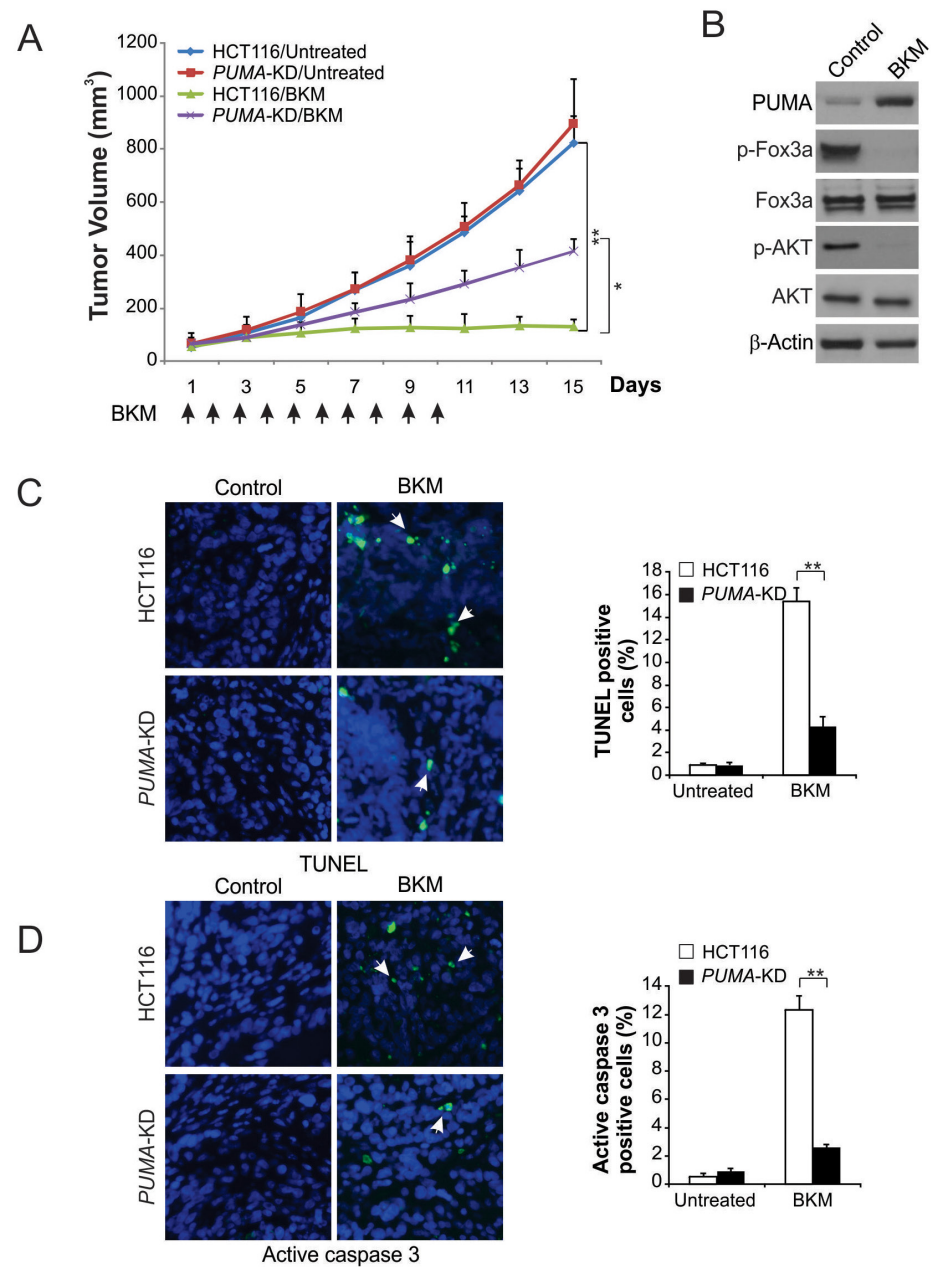
PUMA mediates the antitumor effects of NVP-BKM120 in a xenograft model **(A)** Nude mice were injected s.c. with 4 × 10^6^ parental and *PUMA*-KD HCT116 cells. After 1 week, mice were treated with 40 mg/kg NVP-BKM120 or buffer for 10 consecutive days. Tumor volume at indicated time points after treatment was calculated and plotted (*n*=6 in each group). Arrows indicate NVP-BKM120 injection. **(B)** Parental HCT116 xenograft tumors were treated with 40 mg/kg NVP-BKM120 or the control buffer as in (A) for 4 consecutive days. Indicated protein in representative tumors were analyzed by Western blotting. **(C)** Paraffin-embedded sections of tumor tissues from mice treated as in (B) were analyzed by TUNEL staining. *Left,* representative TUNEL staining pictures; *Right,* TUNEL-positive cells were counted and plotted. **(D)** Tissue sections from (C) were analyzed by active caspase 3 staining. *Left,* representative staining pictures; *Right,* active caspase 3-positive cells were counted and plotted. Results of (A), (C) and (D) were expressed as means ± SD of 3 independent experiments. **, *P*<0.01; *, *P*<0.05.

To determine the role of PUMA in anticancer effects of NVP-BKM120 *in vivo*, nude mice were injected with parental and *PUMA*-KD HCT116 cells to generate xenograft tumor model. After one week, mice with tumor were treated daily with 40 mg/kg NVP-BKM120 or control for 10 consecutive days by oral gavage. For the vehicle treatment, no significantly difference was found between parental and *PUMA*-KD tumors in growth (Figure 6A). The growth of parental tumors was suppressed with NVP-BKM120 treatment by 70-80% (Figure 6A). However, compared to parental tumors, *PUMA*-KD tumors were insensitive to NVP-BKM120 (Figure 6A), indicating that PUMA mediates the anti-tumor effects of NVP-BKM120. In NVP-BKM120-treated tumors, phosphorylation of FoxO3a and expression of PUMA were increased (Figure 6B). Our results showed that compare to control mice, significant apoptosis induction was found in tumor tissues in the mice treated with NVP-BKM120 by TUNEL staining. However, the tumor suppression by NVP-BKM120 treatment was largely abolished in the mice with *PUMA*-KD tumors (Figure 6C), which was also associated with decreased apoptosis detected by active caspase 3 staining (Figure 6D). These findings suggest that PUMA mediated the antitumor effect of NVP-BKM120 *in vivo*.

## DISCUSSION

PI3K inhibitor NVP-BKM120, a well-tolerated agent, showed an effective induction of apoptosis in tumor cells and cancer xenograft models at clinically relevant doses [19, 20]. ALL four isoforms of Class I PI3K (α, β, γ, and δ) are selectively targeted by this oral PI3K inhibitor [8]. NVP-BKM120 has demonstrated proapoptotic, anti-proliferative, and anticancer effects in a variety of cell lines and animal models through cancers-dependent and -independent aberrant PI3K pathway activation [5, 21]. In the current study, we examined the effect of NVP-BKM120 on CRC. Our results demonstrated for the first time that the tumor suppression by NVP-BKM120 is at least in part mediated by the cell autonomous process of apoptosis induction, progressing from AKT inhibition, FoxO3a activation, leading to PUMA induction and onset of mitochondria-dependent apoptosis.

PUMA induction in response to p53 and/or other apoptotic signals, which are associated with mitochondria and induce cell death when overexpressed in different kinds of cell lines, and the activity of apoptotic requires an complete BH3 domain [22]. PUMA has been shown involved in the critical process of tumorgenesis [23]. Previously study indicated that PUMA should be a potential chemotherapeutic target because activated PUMA inhibits tumor growth by restoring cell apoptosis in cancer cells [24]. In addition, PUMA can also be stimulated via a p53-independent way. In non-inflammatory toxin-stimulated conditions such as inflammatory cytokines, deprivation of growth factory and p53-independent PUMA induction can be mediated by various transcription factors such as NF-κB, E2F1, FoxO3a, and p73 [25-27]. PUMA induction strongly promoted cancer cell apoptosis by acting on other Bcl-2 family members, and triggered caspase cascade [28, 29]. These findings suggest that antitumor effect of NVP-BKM120 via p53-independent PUMA induction.

The current study indicates that PUMA-induced by NVP-BKM120 initiates apoptosis via the intrinsic apoptosis pathway in CRC. Several reports showed that PUMA induction plays a key role in apoptosis induction in response to numbers of chemotherapeutic agents, and is likely to be a useful biomarker of chemo-sensitivity [17, 22, 23]. The results demonstrate that the induction of PUMA can be used as a biomarker for predicting response of CRC to NVP-BKM120.

In summary, our results revealed a novel anticancer mechanism of NVP-BKM120 via PUMA-mediated apoptosis. AKT/FoxO3a signaling pathway was involved in NVP-BKM120-induced PUMA expression. Our results indicated PUMA induction can be used as a biomarker for clinical trials testing NVP-BKM120, and can help important implications for the future development and application.

## MATERIALS AND METHODS

### Cell culture and treatment

The human colon cancer cell lines including HCT116, DLD1, HT29, Lim2405, SW480 and LoVo were obtained from American Type Culture Collection. All colon cancer cell lines were cultured in DMEM medium supplemented with 10% newborn calf serum, 100 units/mL penicillin, and 100 μg/mL streptomycin (Invitrogen). The anticancer chemicals used including NVP-BKM120, regorafenib (Selleckchem), and 5-fluoreuracil (5-FU, Sigma) were diluted with DMSO. Constitutively active AKT was obtained from Addgene.

### MTS assay

The indicated cell lines were seeded in 96-well plates at a density of 1×10^4^ cells/well. After overnight incubation, cells were treated with NVP-BKM120 for 72 hours. 3-(4, 5-dimethylthiazol-2-yl)-5-(3-carboxymethoxyphenyl)-2-(4-sulfophenyl)-2H-tetrazolium (MTS) assay was performed using the MTS assay kit (Promega) according to the manufacturer’s instructions. Luminescence was measured with a Wallac Victor 1420 Multilabel Counter (Perkin Elmer). Each assay was conducted in triplicate and repeated three times.

### Real-time reverse transcriptase (RT) PCR

Total RNA was extracted using the TRIzol RNA Kit (Invitrogen) according to the manufacturer’s protocol. One μg of total RNA was used to generate cDNA using SuperScript II reverse transcriptase (Invitrogen). PCR was performed using SsoFasr™ Probes Supermix (Bio-Rad) in a final reaction volume of 20 μl with gene-specific primer/probe sets, and a standard thermal cycling procedure (35 cycles) on a Bio-Rad CFX96™ Real-time PCR System. PUMA and b-actin levels were assessed using TaqMan Gene Expression Real-Time PCR assays. Result was expressed as the threshold cycle (Ct). The relative quantification of the target transcripts was determined by the comparative Ct method (ΔΔCt) according to the manufacturer’s protocol. The 2^-ΔΔCt^ method was used to analyze the relative changes in gene expression. Control experiments were conducted without reverse transcription to confirm that the total RNA was not contaminated with genomic DNA. β-Actin was used as an internal control gene in order to normalize.

### Western blotting

Western blotting was performed as previously described [30], with antibodies for PUMA, Mcl-1 (Abcam), AKT, phospho-AKT, cleaved-caspase 3, cleaved-caspase 9, cleaved-caspase 8, phospho-FoxO3a, FoxO3a, cytochrome oxidase subunit IV (Cox IV), E2F1, p73, Bim, Bcl-2, Bcl-X_L_ (Cell Signaling Technology), cytochrome *c*, β-actin, Noxa (Santa Cruz Biotechnology).

### Apoptosis assays

Apoptosis was analyzed by nuclear staining with Hoechst 33258 (Invitrogen) [31]. Annexin V/propidium iodide (PI) staining was performed using annexin-Alexa 488 (Invitrogen) and PI. Caspase 3/7 activity was detected with a Cell Death Detection ELISA^Plus^ Kit (Roche Molecular Biochemicals, Indianapolis). For analysis of cytochrome *c* release, cytosolic fractions were isolated by differential centrifugation, and probed by Western Blotting for cytochrome c. For colony formation assays, the treated cells were plated in 12-well plates at appropriate dilutions and allowing for cell growth for 2 weeks, followed by crystal violet staining of cell colonies.

### Transfection and siRNA/shRNA knockdown

Cells were transfected with Lipofectamine 2000 (Invitrogen) according to the manufacturer’s instructions. Knockdown experiments were performed 24 hours prior to NVP-BKM120 treatment using 200 pmole of siRNA. The control scrambled siRNA and siRNA for human *FoxO3a* were from Santa Cruz Biotechnology and Thermo Fisher Scientific. For stable transfection a shRNA-expressing plasmid that containing PUMA-targeting sequence (CCTGGAGGGTCATGTACAATCTCTT), or a vector containing a scrambled sequence was transfected into HCT116 cells, followed transfection, cells were plated in 96-well plates in the presence of 5μg/mL puromycin. The protein expression of puromycin-resistant clones was then analyzed by western blotting.

### Chromatin immunoprecipitation (ChIP)

ChIP with FoxO3a antibody (Cell Signaling Technology) was performed using the Chromatin Immunoprecipitation Assay Kit (Millipore) according to the manufacturer’s instructions. The precipitates were analyzed by PCR using primers 5’-GCGCACAGGTGCCTCGGC-3’ and 5’-TGGGTGTGGCCGCCCT-3’.

### Animal tumor experiments

All the experimental procedures were carried out following the guidelines of The People’s Hospital of Liaoning Province and have been approved by the Committee for animal experimentation of The People’s Hospital of Liaoning Province. Parental and *PUMA*-KD HCT116 cells were harvested, and 4 × 10^6^ cells in 0.1 mL of medium were implanted subcutaneously on the back of athymic nude female mice. After tumor growth for 7 days, mice were treated with daily with NVP-BKM120 at 40 mg/kg by oral gavage for 10 consecutive days. Tumor growth was monitored by calipers, and tumor volumes were calculated according to the 12×length×width2. Mice were euthanized when tumors reached ∼1.0 cm^3^ in size. Tumors were dissected and fixed in 10% formalin and embedded in paraffin. TUNEL and active caspase 3 immunostaining was performed on 5 μM paraffin-embedded tumor sections, by using an Alexa Fluor 488-conjugated secondary antibody (Invitrogen) for signal detection.

### Statistical analysis

Statistical analyses were carried out using GraphPad Prism V software. *P* values were calculated by the student’s t-test and were considered significant if p < 0.05. The means ± standard deviation (SD) is displayed in the figures.

## Abbreviations

ChIP: chromatin immunoprecipitation
Cox IV: cytochrome oxidase subunit IV
CRC: colorectal cancer
E2F1: E2F transcription factor 1
FoxO3a: Forkhead Box O3a
5-FU: 5-fluoreuracil
PUMA: p53 upregulated modulator of apoptosis
RT-PCR: reverse transcriptase-PCR
STAT1: signal transducer and activator of transcription 1
TUNEL: terminal deoxynucleotidyl transferase mediated dUTP nick end labeling
